# Frontal deficits and atrophy in a patient with familial encephalopathy with neuroserpin inclusion bodies detected by single-case voxel-based morphometry: a case report

**DOI:** 10.1186/s12883-023-03511-0

**Published:** 2024-01-02

**Authors:** Hideo Handa, Atsuhiko Sugiyama, Tadashi Kaname, Yoko Shigemoto, Noriko Sato, Shigeki Hirano, Yuki Nakagawa, Akiyuki Uzawa, Akiyo Aotsuka, Satoshi Kuwabara

**Affiliations:** 1 Department of Neurology, Graduate School of Medicine, Chiba University, 1-8-1 Inohana, Chuo-ku, Chiba, 260-8677 Japan; 2 Department of Neurology, Chiba Aoba Municipal Hospital, Chiba, Japan; 3 Department of Genome Medicine, National Center for Child Health and Development, Tokyo, Japan; 4 Department of Radiology, National Center Hospital, National Center of Neurology and Psychiatry, Tokyo, Japan

**Keywords:** Familial encephalopathy with neuroserpin inclusion bodies (FENIB), *SERPINI1*, Progressive myoclonic epilepsy (PME), Voxel based morphometry (VBM)

## Abstract

**Background:**

Familial encephalopathy with neuroserpin inclusion bodies (FENIB) is a rare genetic disorder characterized by progressive cognitive decline and myoclonic epilepsy, caused by pathogenic variants of *SERPINI1*. We reported a case of genetically confirmed FENIB with de novo H338R mutation in the *SERPINI1*, in which frontal deficits including inattention and disinhibition, and relevant atrophy in the vmPFC on brain MRI were observed in the early stage of the disease.

**Case presentation:**

A 23-year-old Japanese man presented with progressive inattention and disinhibition over 4 years followed by myoclonic epilepsy. The whole-genome sequencing and filtering analysis showed de novo heterozygous H338R mutation in the *SERPINI1*, confirming the diagnosis of FENIB. Single-case voxel-based morphometry using brain magnetic resonance imaging obtained at the initial visit revealed focal gray matter volume loss in the ventromedial prefrontal cortices, which is presumed to be associated with inattention and disinhibition.

**Conclusion:**

Frontal deficits including inattention and disinhibition can be the presenting symptoms of patients with FENIB. Single-case voxel-based morphometry may be useful for detecting regional atrophy of the frontal lobe in FENIB. Detecting these abnormalities in the early stage of disease may be key findings for differentiating FENIB from other causes of progressive myoclonic epilepsy.

## Background

Familial encephalopathy with neuroserpin inclusion bodies (FENIB) is a rare autosomal dominant genetic disorder that is caused by a genetic defect of *SERPINI1*^1^. *SERPINI1* encodes neuroserpin, also known as proteinase inhibitor 12, which is highly expressed in the neocortex, putamen, and spinal cord, but it is weakly expressed in the cerebellum. Neuroserpin plays an important role in axogenesis, synaptogenesis, and neuroplasticity [1]. Mutated neuroserpin, as observed in H338R and G392E mutations, forms polymers and aggregates in the endoplasmic reticulum, forming the intracytoplasmic inclusion bodies (Collins bodies). Finally, the overloaded neuroserpin inclusion bodies are considered to damage the neurons [2–4].

Although a few case reports on FENIB reported that the frontal area is predominantly affected in the early stage of the disease and other brain regions were involved in the late stage of the disease [2, 5], the other case reports suggested that the clinical presentation of FENIB is indistinguishable from the other causes of progressive myoclonic epilepsies (PMEs, the syndrome that shares epilepsy, cognitive deterioration, and action myoclonus) and that brain magnetic resonance imaging (MRI) in patients with FENIB shows nonspecific brain atrophy. Therefore, it has been suggested that only pathological studies or whole genome sequencing can distinguish this disease from other causes of PME [6–11]. However, previous reports have not fully described the cognitive dysfunction and brain MRI abnormality of early-stage FENIB. Moreover, brain atrophy has only been visually assessed by MRI.

In the present paper, we describe a case of genetically proven FENIB with inattention and disinhibition as early manifestations. Furthermore, we investigated the anatomical abnormalities associated with these clinical features by conducting a single-case voxel-based morphometry analysis.

## Case presentation

**Fig. 1:**
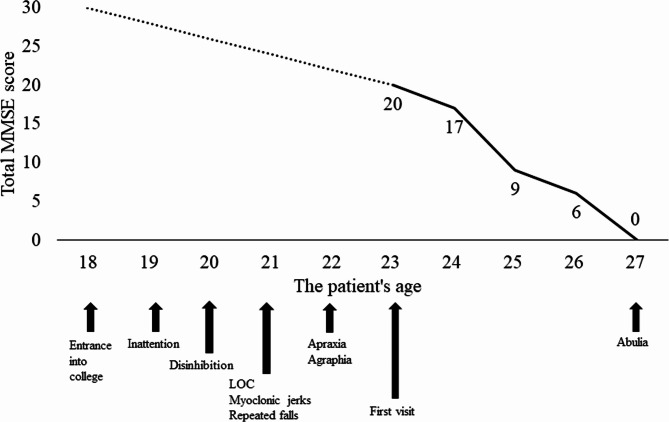
The patient’s clinical course and chronological changes of the Mini-Mental State Examination score. The dotted line, extending from ages 18 to 23, presupposed normal cognitive function at the time when he passed the college entrance examination

A 23-year-old right-handed man with behavioral problems presented to our hospital. He was born at term by normal spontaneous delivery with a birth weight of 3200 g. He had been healthy and achieved good grades until 19 years of age when he became unable to focus on his classes in college. After taking a leave of absence, his behavior further deteriorated (Fig. 1). He shoplifted several times and stole a purse in a public place. When he was 21 years old, he lost consciousness several times and developed myoclonic jerks during his sleep. At 22 years of age, he had agraphia and apraxia (e.g., unable to cut a piece of paper with scissors). His parents were not in a consanguineous marriage and none of his family had neurological disorders.

On examination, he appeared to be restless, impulsive, and distracted. He continuously had action myoclonus on both of his hands. Otherwise, neurological examinations showed normal findings for the cranial nerves, muscle strength, deep tendon reflexes, sensation, cerebellar, and extrapyramidal signs.

**Fig. 2:**
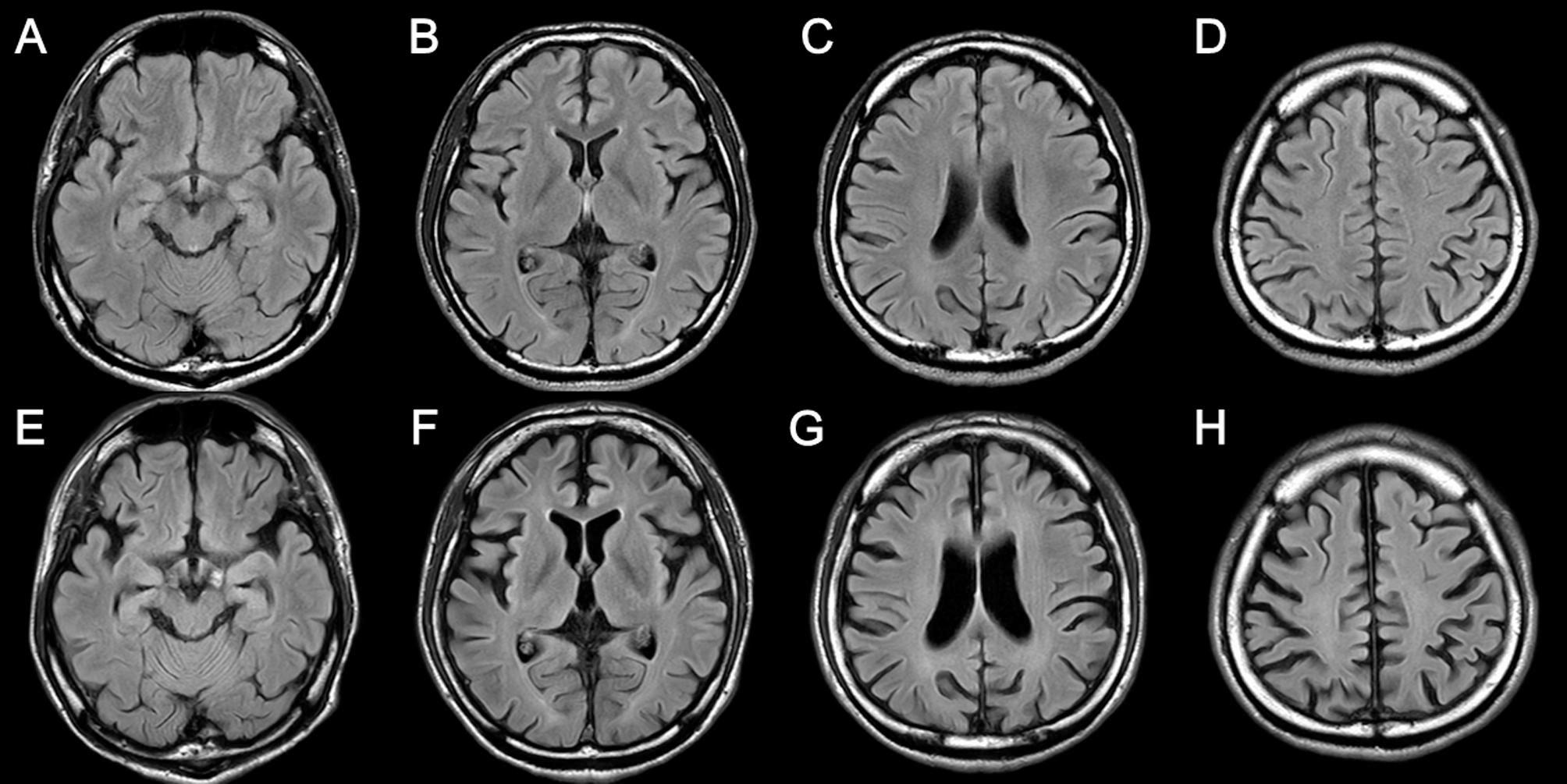
Brain magnetic resonance imaging (MRI) findings. Fluid-attenuated inversion recovery (FLAIR) images of brain MRI obtained at the first visit (23 years old) (**A–D**) showed mild diffuse brain atrophy. The FLAIR images of follow-up brain MRI obtained at 4 years after the initial one (**E–H**) showed more advanced diffuse brain atrophy

He scored 46 out of 100 on Addenbrooke’s Cognitive Examination version III (ACE-III) with the following subscores: attention and orientation, 10/18; memory, 12/26; fluency, 2/14; language, 17/26; and visuospatial skills, 5/16. Electroencephalogram showed generalized intermittent 3–5-Hz spike-and-slow-wave complexes. Brain MRI showed non-significant findings, except for mild diffuse brain atrophy (Fig. 2). N-isopropyl-p-(123I)-iodoamphetamine (IMP) single-photon emission computed tomography (SPECT) revealed widespread hypoperfusion in the cerebral cortices. The whole-exome sequencing and filtering analysis of the patient and his parents identified de novo H338R mutation in the *SERPINI1*. The H338R mutation, previously reported as a pathogenic mutation causing FENIB, confirmed the diagnosis of FENIB [4].

As treatment of the patient’s epileptic seizures, he was prescribed with 800-mg valproic acid daily, which suppressed the epileptic seizures. His cognitive impairment gradually progressed (Fig. 1). At the last follow-up (4 years after the first visit), he became incommunicative. Follow-up MRI showed more advanced diffuse brain atrophy (Fig. 2). IMP SPECT showed diffuse hypoperfusion in the cerebral cortices.

### Brain MRI analysis

**Fig. 3:**
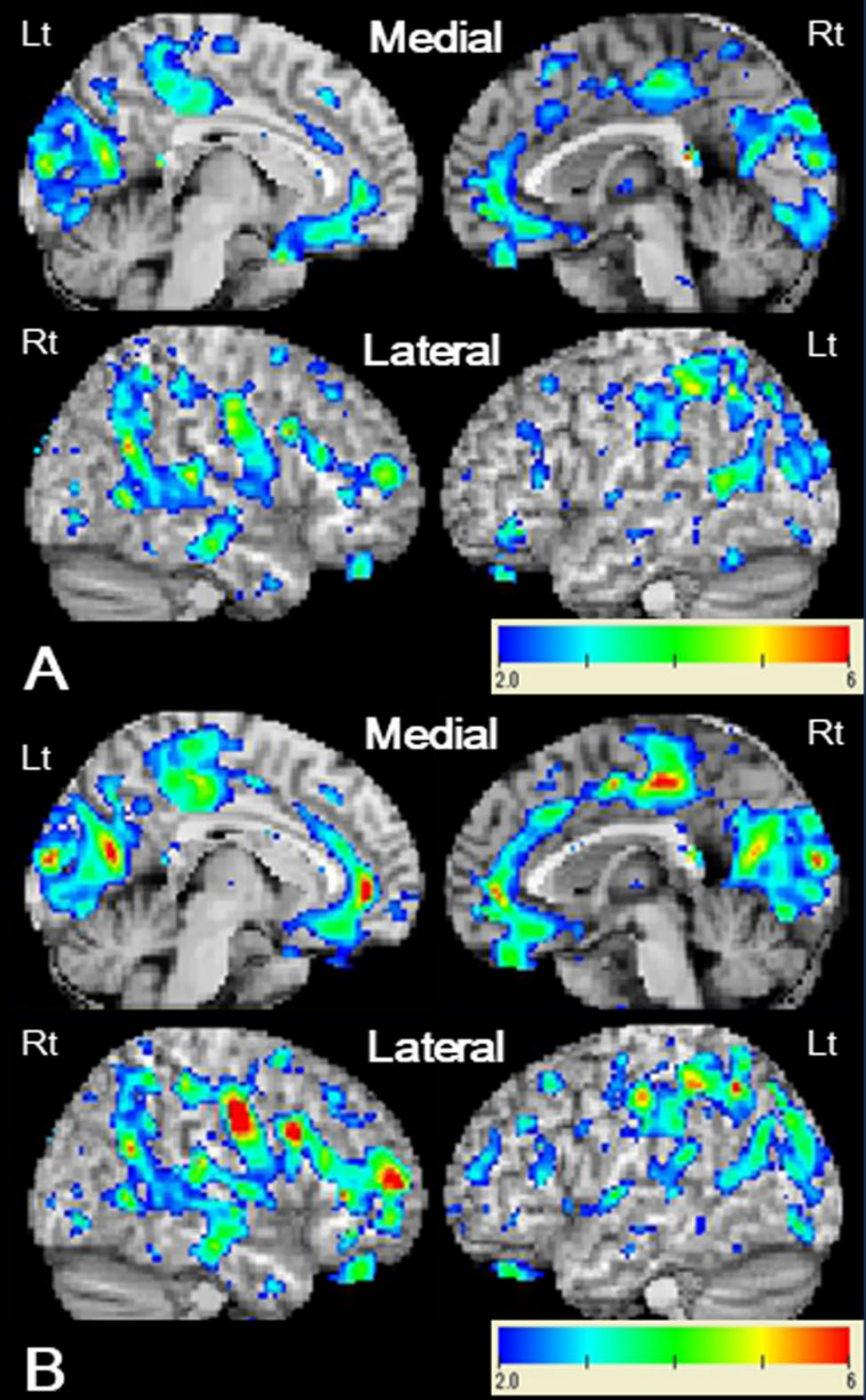
Results of the single-case voxel-based morphometry (VBM) analysis. The VBM analysis using MRI at the first visit (**A**) shows gray matter volume reductions in the ventromedial prefrontal cortices, occipitoparietal cortices on both sides, and posterior part of the temporal lobe on the right side. The VBM analysis using MRI scans obtained at 4 years after the initial one (**B**) demonstrated more severe and extensive gray matter volume reductions in the ventromedial prefrontal cortices, occipitoparietal cortices, and cingulate gyri on both sides, and right temporal gyrus

Single-case voxel-based morphometry (VBM) was performed using 3D T1-weighted images to assess the structural brain changes in the gray matter volume. In our single-case VBM analysis, we used the software “voxel-based specific regional analysis system for Alzheimer’s disease advance” (Eisai, Tokyo, Japan) equipped with SPM8 (The Wellcome Trust Centre for Neuroimaging, Institute of Neurology, University College London, UK) and Diffeomorphic Anatomical Registration using Exponentiated Lie Algebra (DARTEL) based on VBM [12]. To assess the pattern of gray matter volume reduction in the patient, the original control data in the software were replaced with the data of 15 age-matched disease controls (15 men; mean age 23.6 ± 3.0 years; disorder five cases of sleep disorders, four cases of long coronavirus disease, two cases of narcolepsy, two cases of attention-deficit hyperactivity disorder, one case of chronic fatigue syndrome, and one case of anxiety disorder). The segmented gray matter images were compared with the mean and standard deviation of the images of the 15 disease controls using voxel-by-voxel Z-score analysis with voxel normalization to global mean intensities. The Z-score was calculated as follows: Z-score = ([control mean] − [individual value]) / (control standard deviation). A Z-score > 2 was defined as significant. Single-case VBM analysis using MRI at the first visit showed gray matter volume reductions in the ventromedial prefrontal cortices (vmPFC), occipitoparietal cortices on both sides, and the posterior part of the temporal lobe on the right side (Fig. 3). The single-case VBM analysis using MRI scans obtained at 4 years after the initial one demonstrated more severe and extensive gray matter volume reductions in the vmPFC, occipitoparietal cortices, cingulate gyri, and right temporal gyrus (Fig. 3).

To predict the patient’s brain age, the support regression model implemented in the LIBSVM (http://www.csite.ntu.edu.tw/~cjlin/libsvm/) toolbox with a linear kernel and default set of parameters was used (i.e., in the LIBSVM: *C* = 1, *v* = 0.5). The details of the analytical method were described in a previous study [13]. For the regression model, the chronological age was considered the dependent variable, whereas the principal components derived from the gray matter voxel intensities were considered independent variables. Brain-age prediction analysis using the MRI scan obtained at the first visit revealed a predicted brain age and brain-predicted age difference (brain-PAD: predicted brain age–chronological age) of 60.06 and 36.63, respectively. Brain-age prediction analysis using the MRI scan obtained at 4 years after the initial one revealed a predicted brain age and brain-PAD of 80.87 and 53.58, respectively.

## Discussion and conclusions

In this paper, we reported a case of genetically confirmed FENIB with de novo H338R mutation in the *SERPINI1*, in which frontal deficits including inattention and disinhibition and relevant atrophy in the vmPFC on brain MRI were observed in the early stage of the disease. Our study findings suggest that the frontal lobe, including vmPFC, is preferentially involved in the early stage of FENIB and that the detection of frontal lobe dysfunction in the early stage may be helpful in differentiating FENIB from other causes of PME. Note that, to the best of our knowledge, this is the first report of focal volume loss detected by MRI in a case of FENIB.

Clinicians should be aware that patients with FENIB developed frontal deficits including inattention and disinhibition at the early stage of the disease. Similar to our case, multiple cases have presented with frontal deficits including inattention or hyperactivity at the initial phase [5, 7–11]. Additionally, pediatric patients can be misdiagnosed with attention-deficit hyperactivity disorder (ADHD) [10] because the initial symptoms of FENIB, such as inattention and hyperactivity, can be difficult to differentiate from those of ADHD. These characteristic frontal deficits can be explained by the autopsy findings of individuals with FENIB, which revealed that the most of the frontal areas were considerably atrophic and abundant in Collins bodies [5, 6]. Frontal deficits have not been reported as the characteristic portents in other causes of PMEs, including Lafora disease, Unverricht-Lundborg disease, sialidosis, myoclonus epilepsy associated with ragged-red fibers, and neuronal ceroid lipofuscinosis [14]. Therefore, the initial frontal deficits might be helpful to differentiate FENIB from other PMEs.

Volumetric MRI analysis may detect imaging abnormalities associated with frontal deficits in FENIB. In the present case, although the initial MRI based on the visual assessment only showed mild generalized brain atrophy and the visual assessment of follow-up MRI showed more advanced generalized brain atrophy, single-case VBM detected atrophy of the regional gray matter of vmPFC in the early stage of disease and a more generalized gray matter atrophy in the late stage of disease. Previous studies hinged only on the visual assessment and those studies may have missed this finding of vmPFC atrophy. Single-case VBM also detected gray matter atrophy in the posterior brain regions, including the occipitoparietal and posterior temporal lobes in the current case, and this pattern of gray matter atrophy is similar to that of previously reported case of posterior cortical atrophy [15, 16]. These areas are included in the ventral and dorsal visual pathways and may be associated with the deficits in visuospatial skills detected in ACE-III in this case.

Predicted brain age and brain-PAD (predicted brain age–chronological age) has potential as a fully automated imaging index that can be used to assess brain atrophy level and degenerative process progression in patients with FENIB. Recent advances in machine learning have allowed the prediction of the age of an individual’s brain image using a regression model [17]. This “neuroimaging-based brain-age prediction” has recently been applied to neurodegenerative disorders and has helped the early diagnosis of Alzheimer’s disease and the prediction of the conversion of cases of mild cognitive impairment to Alzheimer’s disease [17]. In Alzheimer’s disease, brain-PAD increased along with the Alzheimer’s disease severity, and it was correlated well with neuropsychological scores [18]. In our case, brain-age prediction analysis using an MRI scan obtained at the first visit showed a brain-PAD increase of 36.63 years, which is much greater than the 10-year brain-PAD increase reported in Alzheimer’s disease patients [17]. Moreover, the analysis using MRI obtained at 4 years later showed an even greater increase in brain-PAD of 53.58 years, reflecting well the rapid progression of cognitive dysfunction in the present case.

In conclusion, frontal deficits including inattention and disinhibition can be the presenting symptoms in patients with FENIB. Volumetric analysis of brain MRI, such as single-case VBM, may be useful for detecting regional atrophy of the frontal lobe in FENIB. Detecting these abnormalities in the early stage of the disease can be potential key findings for differentiating FENIB from other causes of PME.

## Data Availability

Not applicable.

## List of abbreviations

PME: Progressive myoclonic epilepsy
MRI: Magnetic resonance imaging
VBM: Voxel-based morphometry
vmPFC: Ventromedial prefrontal cortices
ACE-III: Addenbrooke’s Cognitive Examination
IMP: Iodoamphetamine
SPECT: Single-photon emission computed tomography
DARTEL: Diffeomorphic Anatomical Registration using Exponentiated Lie Algebra
Brain-PAD: Brain-predicted age difference
